# Confirmatory testing of primary aldosteronism with saline infusion test and LC-MS/MS

**DOI:** 10.1530/EJE-20-0073

**Published:** 2020-10-20

**Authors:** Carmina Teresa Fuss, Katharina Brohm, Max Kurlbaum, Anke Hannemann, Sabine Kendl, Martin Fassnacht, Timo Deutschbein, Stefanie Hahner, Matthias Kroiss

**Affiliations:** 1 Division of Endocrinology and Diabetes, Department of Internal Medicine I, University Hospital, University of Würzburg, Würzburg, Germany; 2 Central Laboratory, Core Unit Clinical Mass Spectrometry, University Hospital Würzburg, Würzburg, Germany; 3 Institute of Clinical Chemistry and Laboratory Medicine, University Medicine Greifswald, Greifswald, Germany; 4 Department of Medicine IV, University Hospital Munich, Ludwig-Maximilians-Universität München, Munich, Germany

## Abstract

**Objective:**

Saline infusion testing (SIT) for confirmation of primary aldosteronism (PA) is based on impaired aldosterone suppression in PA compared to essential hypertension (EH). In the past, aldosterone was quantified using immunoassays (IA). Liquid chromatography tandem mass spectrometry (LC-MS/MS) is increasingly used in clinical routine. We aimed at a method-specific aldosterone threshold for the diagnosis of PA during SIT and explored the diagnostic utility of steroid panel analysis.

**Design:**

Retrospective cohort study of 187 paired SIT samples (2009–2018). Diagnosis of PA (*n* = 103) and EH (*n* = 84) was established based on clinical routine workup without using LC-MS/MS values.

**Setting:**

Tertiary care center.

**Methods:**

LC-MS/MS using a commercial steroid panel. Receiver operator characteristics analysis was used to determine method-specific cut-offs using a positive predictive value (PPV) of 90% as criterion.

**Results:**

Aldosterone measured by IA was on average 31 ng/L higher than with LC-MS/MS. The cut-offs for PA confirmation were 54 ng/L for IA (sensitivity: 95%, 95% CI: 89.0–98.4; specificity: 87%, 95% CI: 77.8–93.3; area under the curve (AUC): 0.955, 95% CI: 0.924–0.986; PPV: 90%, 95% CI: 83.7–93.9) and 69 ng/L for LC-MS/MS (79%, 95% CI: 69.5–86.1; 89%, 95% CI: 80.6–95.0; 0.902, 95% CI: 0.857–0.947; 90%, 95% CI: 82.8–94.4). Other steroids did not improve SIT.

**Conclusions:**

Aldosterone quantification with LC-MS/MS and IA yields comparable SIT-cut-offs. Lower AUC for LC-MS/MS is likely due to the spectrum of disease in PA and previous decision making based on IA results. Until data of a prospective trial with clinical endpoints are available, the suggested cut-off can be used in clinical routine.

## Introduction

Primary aldosteronism (PA) is characterized by an autonomous aldosterone secretion leading to sodium retention, arterial hypertension and hypokalemia (1). PA is the most common endocrine cause of secondary hypertension, with a prevalence between 5 and 13% in patients with arterial hypertension (1, 2, 3). The disease is associated with an increased cardiovascular risk and renal complications (2, 4, 5). Whereas unilateral oversecretion of aldosterone can be cured by adrenal surgery, patients with bilateral disease receive life-long treatment with mineralocorticoid antagonists (1). Therefore, correct diagnosis and subsequent subtype differentiation are crucial for adequate clinical management. The current Clinical Practice Guideline of the Endocrine Society recommends screening by determination of the aldosterone/renin ratio (ARR) (1). Confirmatory testing can be accomplished by using the saline infusion test (SIT), captopril challenge, fludrocortisone suppression, or oral sodium loading (1). All of those tests differ regarding their reported sensitivity, specificity, and reliability; clear-cut evidence for one optimal confirmatory test is still lacking (6, 7, 8, 9).

Local expertise, costs, patient compliance and laboratory routine guide the choice of the testing procedure (1). Among the available tests, SIT represents one of the most widely used ones which is most likely explained by its simplicity, safety profile, and cost effectiveness (10, 11). Notably, however, cut-off values to rule out PA by SIT are hampered by liabilities of currently used immunoassays (IA), which show low inter-assay agreement particularly in the low range of aldosterone concentration (12, 13, 14). This appears to be caused (at least in part) by cross-reactivity with other compounds and metabolites (15, 16, 17), particularly in patients with impaired renal function (18).

Liquid chromatography tandem mass spectrometry (LC-MS/MS) has been introduced into the clinical routine analysis of steroid hormones (19, 20, 21) due to its higher specificity and is increasingly used for the diagnosis of adrenal diseases (22, 23, 24). Aldosterone concentrations measured with LC-MS/MS are usually lower than those measured by most IAs (15, 17, 25, 26, 27, 28). Guo *et al.* recently proposed a LC-MS/MS-specific cut-off for aldosterone during fludrocortisone suppression testing (28). Regarding SIT, however, LC-MS/MS-derived counterparts are still lacking.

The current study aims to establish LC-MS/MS-specific threshold values for aldosterone during SIT and to determine their diagnostic accuracy for the presence of PA in a cohort of 187 SIT performed under standardized conditions at a single tertiary referral center.

## Subjects and methods

### Study design, participants and saline infusion testing

We retrospectively evaluated 236 consecutive patients who underwent SIT for suspected PA at the University Hospital Würzburg between 2009 and 2018. The sample size was hence determined by the availability of biomaterial within this time frame. The investigation was approved by the Ethics committee of the University of Würzburg (20190123 03). SIT was carried out as confirmatory test in patients with suspected PA due to elevated ARR (>20) using a standardized protocol. Before testing, mineralocorticoid antagonists were discontinued for 4 weeks and antihypertensive medication was adapted allowing only calcium channel blockers (e.g. verapamil) and/or alpha receptor antagonists (e.g. urapidil, doxazosin) for at least 1 week. SIT was carried out in a recumbent position in the morning between 08:00 and 10:00 h . Potassium supplementation was administered to avoid hypokalemia during the test. Patients received 2 L of 0.9% saline solution intravenously over 4 h. Immediately before saline infusion, as well as after 4 h, serum/plasma samples were taken for measurement of aldosterone, renin and potassium and afterwards stored at -80°C.

**Figure 1:**
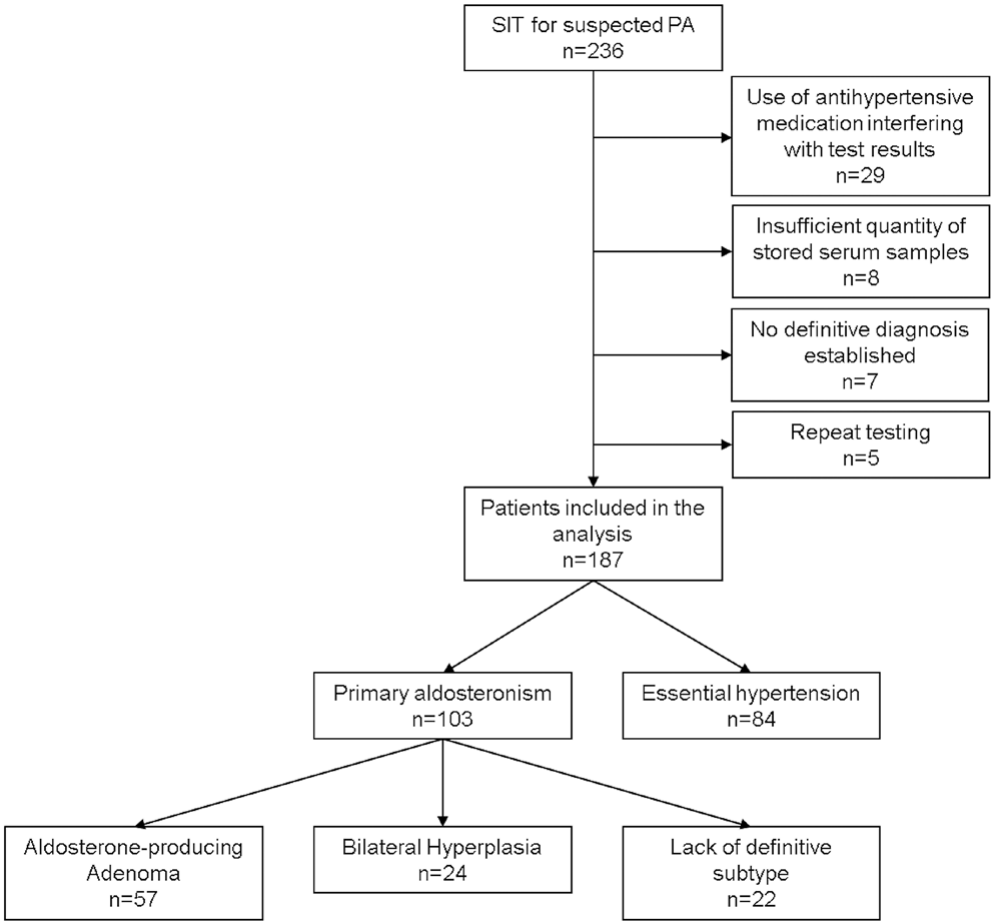
Study flow: 236 patients underwent saline infusion testing (SIT) for suspected primary aldosteronism (PA). 187 patients were included in the final analysis. Unknown = subtype of PA not known due to unsuccessful adrenal vein sampling or patient’s refusal to undergo adrenal vein sampling or surgery.

Forty-nine patients were excluded from the study for the following reasons (Fig. 1): (i) use of antihypertensive medication (beta-blockers, angiotensin-converting enzyme inhibitors, angiotensin receptor blockers) that possibly interferes with test results (*n* = 29), (ii) insufficient quantity of stored serum samples (*n* = 8), (iii) initial testing repeated (with inconclusive results) (*n* = 5), or (iv) no definitive diagnosis after initial workup (*n* = 7). The clinical characteristics of the 187 included patients are given in Table 1.

**Table 1:** Clinical characteristics of study cohort. Data are given as mean ± s.d., *n* (%) or median (interquartile range). Serum aldosterone (ALD) concentration was measured after adaptation of blood pressure medication for SIT. Comparisons were performed with Pearson chi-square for categorical variables, *t*-test for mean comparison of normally distributed data, Mann–Whitney *U*-test for non-normally distributed numerical variables.

	PA	EH	*P*-value	APA	BAH	*P*-value
*n*	103	84		56	24	
Age, years	53 ± 12	52 ± 13	0.409	52 ± 11	52 ± 9	0.900
Male, *n* (%)	66 (64%)	28 (33%)	**<0.001**	34 (61%)	16 (67%)	0.614
BMI, kg/m²	29.1 (21.1–32.5)	26.1 (23.9–29.2)	**<0.001**	28.4 (25.4–31.2)	32.4 (28.1–36.0)	**0.002**
Systolic BP, mmHg	158 (143–168)	150 (140–163)	**0.003**	155 (143–165)	160 (150–180)	0.408
Diastolic BP, mmHg	92 (85–98)	85 (80–92)	**0.004**	93 (85–100)	92 (85–97)	0.505
Diabetes	21 (20.4%)	4 (4.8%)	**0.002**	10 (17.9%)	6 (25.0%)	0.464
Coronary heart disease	5 (4.9%)	2 (2.4%)	0.375	2 (3.6%)	2 (8.3%)	0.370
Stroke	5 (4.9%)	2 (2.4%)	0.375	2 (3.6%)	2 (8.3%)	0.370
Renal insufficiency	8 (7.85)	2 (2.4%)	0.103	3 (5.4%)	2 (8.3%)	0.614
Sleep apnea syndrome	6 (5.8%)	3 (3.6%)	0.474	1 (1.8%)	3 (12.5%)	0.103
Potassium, mmol/L	3.9 (3.4–4.1)	4.3 (4–4.5)	**<0.001**	3.7 (3.3–4.0)	3.9 (3.7–4.1)	**0.030**
Potassium substitution	56 (54.4%)	10 (11.9%)	**<0.001**	36 (64.3%)	13 (54.1%)	**0.018**
Serum ALD conc., ng/L	237 (153–360)	90 (57–139)	**<0.001**	214 (157–356)	259 (139–384)	0.741
Plasma renin conc., ng/L	3.1 (1.7–5.1)	3.8 (2.1–7.1)	**0.014**	3.5 (2.0–5.5)	3.1 (1.6–4.2)	0.404

BP, blood pressure; conc., concentration.

Diagnosis of PA as well as subtype was determined according to the Endocrine Society Practice Guideline (1) taking into account clinical presentation, results of SIT by using IA in clinical routine, preoperative imaging, adrenal vein sampling (AVS), adrenal pathology, as well as therapeutic outcome (i.e. of surgical or medical treatment). An aldosterone cut-off at 4 h after saline infusion of 50 ng/L measured by IA was used to confirm presence of PA in patients with positive screening criteria. Records of patients with an aldosterone concentration between 37 and 70 ng/L (*n* = 38) were reviewed independently by two experienced endocrinologists to determine diagnosis of PA. Cases, in which both endocrinologists differed in their assessment, were discussed by a broader panel of endocrinologists. If no consensus regarding final diagnosis could be reached, patients were excluded from the analysis (*n* = 7). In patients with PA willing to undergo surgery, AVS without cosyntropin was performed to differentiate unilateral from bilateral PA (diagnostic criteria during AVS: selectivity index ≥ 2, lateralization index ≥ 4:1, or ≥ 3:1** and** contralateral suppression).

### Routine measurements of aldosterone and renin by immunoassay

All samples were measured at the clinical laboratory of the Department of Endocrinology and Diabetes of the University Hospital Würzburg. Until September 2014 (*n* = 81, 43.3%), serum aldosterone was determined by Coat-a-Count^®^ RIA ( Siemens), and plasma renin concentration with a Renin III Generation RIA (Cisbio). Starting in October 2014 (*n* = 106, 56.7%) serum aldosterone and plasma renin concentrations were analyzed – after a comprehensive cross-validation – by an automated chemiluminescence immunoassay (CLIA, iSYS, Immuno Diagnostic Systems).

### LC-MS/MS-based measurement of aldosterone and additional gluco-/mineralocorticoids

Measurement of aldosterone in SIT samples was performed in October 2019 by LC-MS/MS using a Sciex 6500+ QTRAP (SCIEX, Framingham, USA) MS-system linked with an Agilent 1290 UHPLC-system (G4226A autosampler, InfinityBinPump, G1316C column-oven, G1330B thermostat) as described previously (23). Analysis was performed with the MassChrom-Steroids in Serum kit (Chromsystems, Gräfelfing) and corresponding isotope standards according to the manufacturer’s instruction. After off-line solid phase extraction of 500 µL serum, 15 µL of the eluted sample were used for analysis. Concentrations were calculated with Analyst Software (1.6.3) via 6 point calibration and 1/x weighting. Lower limits of quantification (LLOQ): aldosterone (10 ng/L), cortisol (0.15 µg/dL), cortisone (0.15 µg/L), corticosterone (0.175 µg/L), 11-deoxycorticosterone (0.023 µg/L), 11-deoxycortisol (0.03 µg/L), 21-deoxycortisol (0.027 µg/L), dihydrotestosterone (42 ng/L), androstenedione (0.022 µg/L), progesterone (0.03 µg/L), dehydroepiandrosterone (0.229 µg/L), dehydroepiandrosteronesulfate (2.44 µg/dL), estradiol (20 ng/L), testosterone (0.005 µg/L) and 17a-hydroxyprogesterone (0.04 µg/L). Furthermore, 18-hydroxycorticosterone was ascertained to be base line separated from aldosterone (Supplementary Fig. 1, see section on supplementary materials given at the end of this article). Correctness of measurements was monitored by commercial quality controls and periodic participation in ring trials.

### Statistical analysis

Statistical analysis was performed using SPSS version 25 (IBM Corp.) and MedCalc version 19.3. Aldosterone values below LLOQ (<10 ng/L for LC-MS/MS, <37 ng/L for IA) were replaced by the following formula using Excel 2010 (Microsoft): c = LLOQ/√2 (random number between 0.75 and 1.5) as previously described (29). Clinical characteristics are presented as means ± standard deviation for normally distributed parameters and medians and interquartile range for non-normally distributed variables. Comparisons were performed with Pearson Chi-Square for categorical variables, *t*-test for mean comparison of normally distributed data, Mann-Whitney*U*-test for non-normally distributed numerical variables, and Wilcoxon test for paired samples. For comparison of different aldosterone assays, Bland-Altman analysis was performed. Correlations were tested both by Pearson and Spearman test. To determine LC-MS/MS specific cut-offs, receiver operating characteristic (ROC) curves, positive/negative likelihood ratios and predictive values were calculated. We decided to use a positive predictive value of 90% as the criterion for confirmation of PA. The prevalence of PA in our cohort is 55% and hence typical for a tertiary reference center. A *P*-value <0.05 was considered statistically significant. To analyze relative changes of different steroids during SIT we calculated fold changes using the following equation:


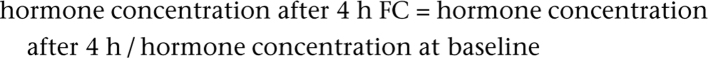


To evaluate the usefulness of steroid profiles for differentiation of unilateral and bilateral PA, principal component analysis (PCA) and partial least square – discriminant analysis (PLS-DA) were performed using MetaboAnalyst 4.0, as previously described (30).

## Results

### Clinical characteristics

Out of 187 patients included into the study, 103 patients were diagnosed with PA, whereas 84 patients were diagnosed with EH (Fig. 1 and Table 1). Patients with PA were more often male, showed significantly higher BMI and blood pressure, higher prevalence of diabetes, lower potassium, higher aldosterone concentrations, and lower renin levels compared to EH (Table 1). No significant differences between the two groups were observed regarding age and comorbidities such as coronary heart disease, stroke, sleep apnea syndrome, and renal insufficiency (Table 1). Subtype differentiation of patients with PA was available for 80 patients. Characteristics of patients with APA and BAH are shown in Table 1. We furthermore retrospectively applied the PASO outcome criteria (31): 46 patients underwent adrenalectomy, 31 had sufficient follow- up (6–102 months). Out of these, 13 had complete, 14 had partial and 4 had absent clinical success. Twenty-eight had complete biochemical success and three absent biochemical success.

### Comparison of radioimmunometric assay and chemiluminescence immunoassay

Over the course of the study, two different IAs were used for aldosterone measurement (RIA, *n* = 81 (43%); CLIA, *n* = 106 (57%)). Table 2 shows aldosterone concentrations during SIT measured by either RIA or CLIA. Aldosterone concentrations from both tests were similar in patients with EH, APA and BAH before and after saline infusion. When cases of APA and BAH were combined, median aldosterone concentration after saline infusion was significantly different (171 ng/L by RIA vs 120 ng/L by CLIA, *P* = 0.027). As expected, patients with PA showed significantly higher aldosterone concentrations during SIT than patients with EH, and this was true for both assays. No significant differences in aldosterone could be detected between patients with APA and BAH.

**Table 2:** Comparison of different immunoassays. Median aldosterone concentration before (0 h) and after (4 h) saline infusion by RIA (*n* = 81) and chemoluminescence assay (CLIA, *n* = 106). Data are given as median and interquartile range. PA subtypes (APA and BAH) were not definitively determined in 22 patients with PA.

	*n*	Aldosterone (ng/L) during the SIT					
		0 h			4 h		
		Coat-a-count® RIA	IDS-iSYS aldosterone® CLIA	*P*-value	Coat-a-count® RIA	IDS-iSYS aldosterone® CLIA	*P*-value
EH	84	92 (71–131)	86 (44–146)	0.414	33 (25–48)	37 (37–40)	0.195
PA	103	210* (147–313)	265* (158–384)	0.235	120* (80–190)	171* (101–246)	**0.027**
APA	57	192 (146–367)	269 (183–376)	0.092	154 (77–191)	175 (119–329)	0.109
BAH	24	153 (139–292)	276 (157–387)	0.290	111 (79–154)	171 (98–242)	0.108

*P < 0.001 vs EH (Mann-Whitney*U*-test).

APA, aldosterone-producing adenoma (RIA: *n* = 23, CLIA: *n* = 34); BAH, bilateral adrenal hyperplasia (RIA: *n* = 9, CLIA: *n* = 15); EH, essential hypertension (RIA: *n* = 37, CLIA: *n* = 47); PA, primary aldosteronism (RIA: *n* = 44, CLIA: *n* = 59).

### Comparison of immunoassays and LC-MS/MS

**Figure 2:**
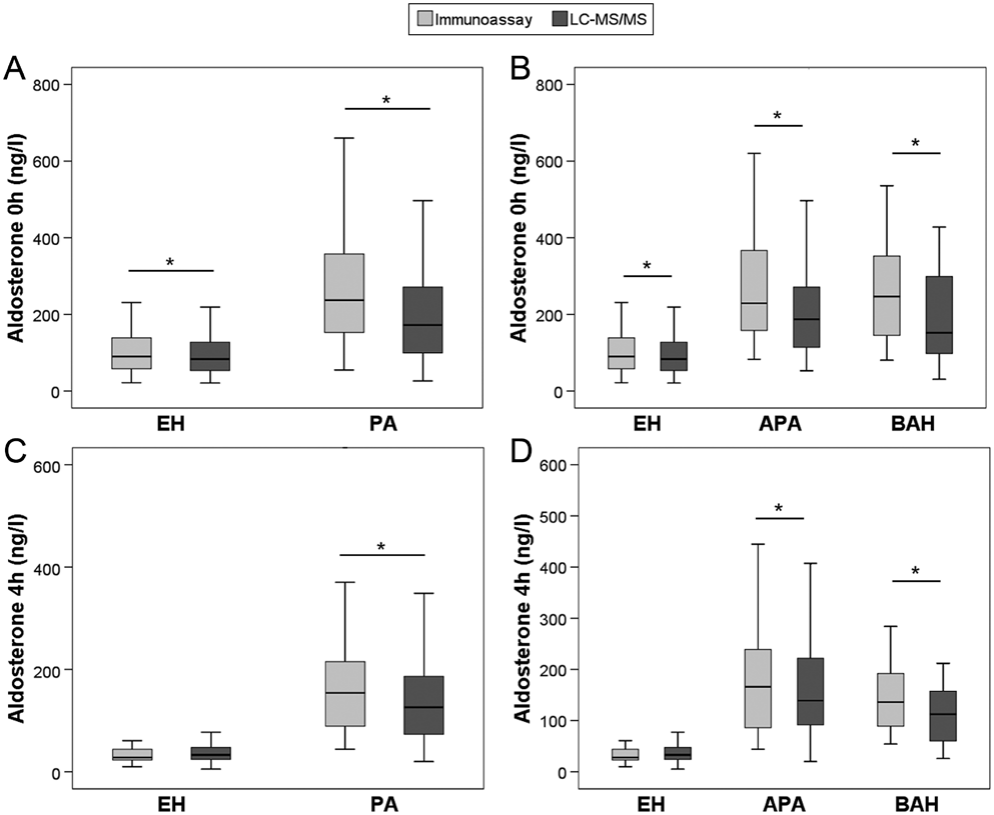
Aldosterone concentrations measured by immunoassay and LC-MS/MS during saline infusion testing in patients with essential hypertension (EH, A, B, C and D), primary aldosteronism (PA, A and C), aldosterone-producing adenoma (APA, B and D) and bilateral adrenal hyperplasia (BAH, B and D) before (0 h, A and B) and after (4 h, C and D) saline infusion. *P < 0.01 (Wilcoxon test).

**Figure 3:**
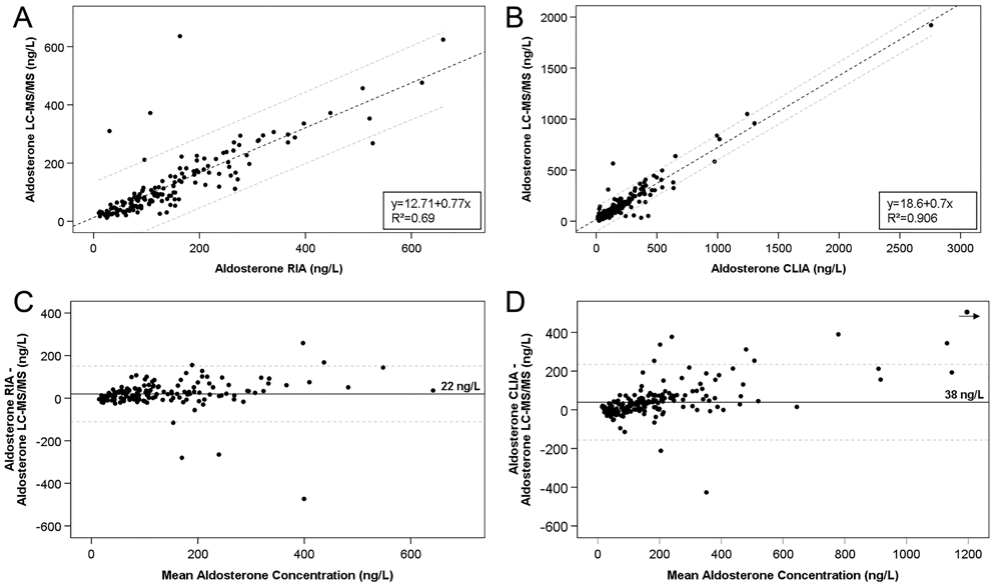
(A and B) Scatter plot of aldosterone concentrations measured by RIA (*n* = 162), CLIA (*n* = 212) and LC-MS/MS. Dashed lines: 95% confidence interval. (A) RIA: Spearman correlation coefficient 0.878 (*P* < 0.001), Pearson r coefficient 0.833 (*P* < 0.001). (B) CLIA: Spearman correlation coefficient 0.877 (*P* < 0.001), Pearson r coefficient 0.952 (*P* < 0.001). (C and D) Bland-Altman analysis of all aldosterone measurements by RIA (C, *n* = 162), CLIA (D, *n* = 212) and LC-MS/MS. Continuous line: mean difference, dashed lines: 95% limits of agreement. For better visualization, x- and y-axes were cut at 1200 ng/L, excluding one single data point from the plot as marked by the dot and arrow in panel D.

Similar to RIA and CLIA, aldosterone concentrations during SIT determined by LC-MS/MS were significantly higher in PA than in EH (172 vs 83 ng/L before and 126 vs 33 ng/L after saline infusion; both *P* < 0.001). In contrast, aldosterone levels at both time points did not differ significantly between patients with APA and BAH (Table 3). Only two patients presented with aldosterone after saline infusion below the LC-MS/MS LLOQ of 10 ng/L in comparison to 54 patients with values below the IA LLOQ of 37 ng/L (all EH). Median aldosterone concentrations measured by LC-MS/MS were significantly lower compared to IA (*P* < 0.01) for baseline samples in EH, PA, APA and BAH, Fig. 2A, B and Table 3; *P* < 0.01 for post-infusion samples of PA, APA, and BAH, Fig. 2C and D). Similar results were obtained, when comparing patients measured by RIA or CLIA to LC-MS/MS (Supplementary Figs 2 and 3). IA and LC-MS/MS measurements of aldosterone showed good linear correlation (Spearman correlation coefficient 0.876, Pearson r coefficient 0.930, both *P* < 0.001) independent of the IA used (RIA/CLIA) (Fig. 3). Bland–Altman plot demonstrated IA-based aldosterone values to be on average 31 ng/L higher than LC-MS/MS (data not shown) with slightly lower deviation of RIA (22 ng/L) compared to CLIA (38 ng/L) (Fig. 3).

**Table 3:** Aldosterone concentrations during saline infusion testing measured by immunoassay and LC-MS/MS. Median aldosterone concentration before (0 h) and after (4 h) saline infusion measured by immunoassay and LC/MS-MS. PA subtypes (APA and BAH) were not definitively determined in 22 patients with PA.

	*n*	Aldosterone (ng/L) during the SIT			
		0 h		4 h	
		Immunoassay	LC-MS/MS	Immunoassay	LC-MS/MS
EH	84	90 (57–139)	83 (53–128)	37 (30–44)	33 (24–48)
PA	103	237* (153–360)	172* (99–271)	154* (89–218)	126* (72–187)
APA	57	229 (156–367)	187 (113–280)	166 (85–242)	139 (90–223)
BAH	24	247 (142–368)	152 (97–301)	136 (87–194)	113 (59–159)

*P < 0.001 vs EH (Mann–Whitney *U*-test).

### Determination of an LC-MS/MS-specific aldosterone threshold

**Figure 4:**
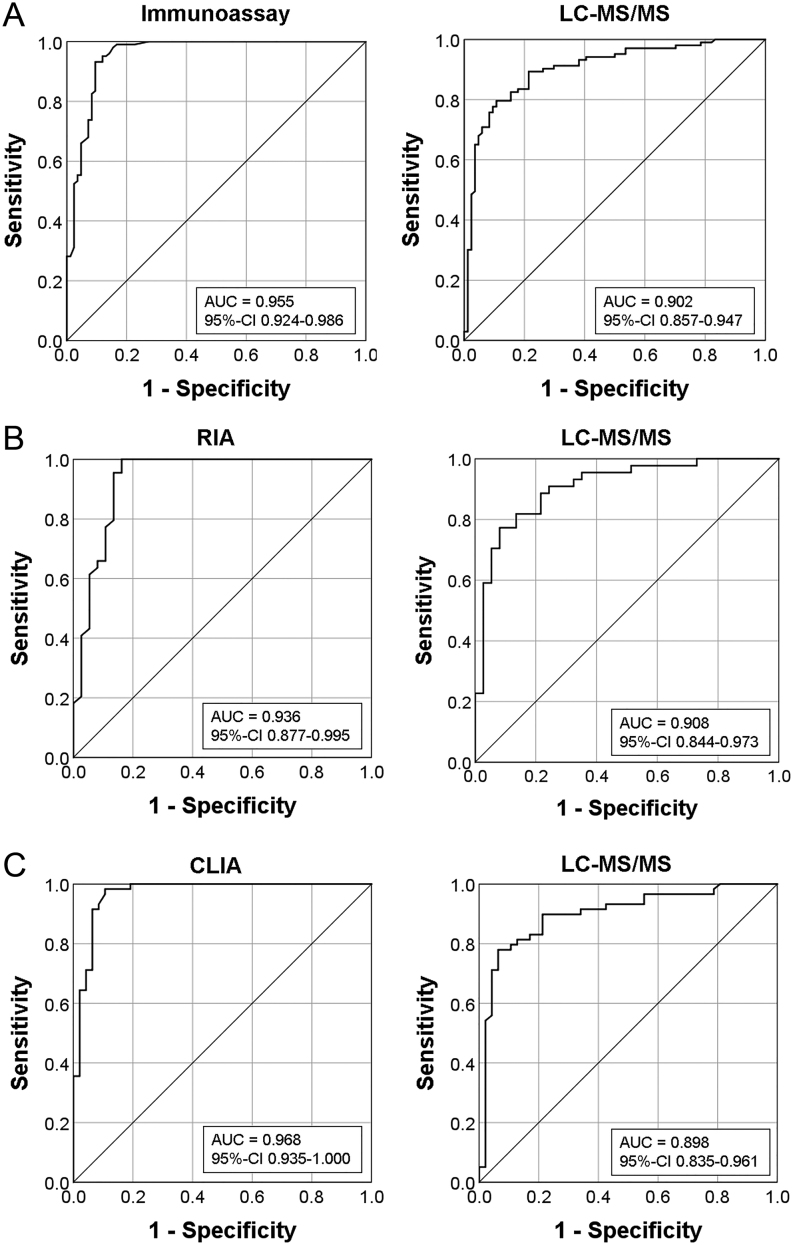
Receiver operating characteristics (ROC) curve for aldosterone concentrations after saline infusion measured by immunoassay and LC-MS/MS for detection of primary aldosteronism. AUC,area under the curve. *P* < 0.001 for both ROC curves.

The aldosterone cut-off was determined by ROC analysis of post SIT aldosterone for diagnosis of PA (Fig. 4). Sensitivity, specificity, likelihood ratios and predictive values of different aldosterone cut-offs are given in Tables 4 and 5. Instead of calculating the frequently used Youden’s index that equally weighs sensitivity and specificity, we decided to use a positive predictive value of 90% as criterion. This approach led to an optimal aldosterone cut-off of 54 ng/L for IA and 69 ng/L for LC/MS-MS, respectively (Tables 4 and 5). Application of the aldosterone cut-off of 69 ng/L for LC-MS/MS led to a misclassification of 31 out of 187 patients (16.6%): nine patients with EH were falsely classified as PA, whereas in 22 patients diagnosis of PA (APA: *n* = 9, BAH: *n* = 8, unknown subtype: *n* = 5) would have been missed. In contrast, only five patients were falsely classified as EH instead of PA, using an aldosterone cut-off of 54 ng/L for IA.

**Table 4:** Sensitivity and specificity of different aldosterone cut-offs after saline infusion measured by immunoassay.

ALD, ng/L	TP, *n*	FP, *n*	TN, *n*	FN, *n*	SN (95% CI)	SP (95% CI)	+LR (95% CI)	-LR (95% CI)	+PV (95% CI)	-PV (95% CI)
>49	102	14	70	1	99.0 (94.7–100.0)	83.3 (73.6–90.6)	5.9 (3.7–9.6)	0.01 (0.002–0.08)	87.9 (81.9–92.2)	98.6 (90.9–99.8)
>50	101	13	71	2	98.1 (93.2–99.8)	84.5 (75.0–91.5)	6.3 (3.8–10.5)	0.02 (0.006–0.09)	88.6 (82.5–92.8)	97.3 (90.0–99.3)
>53	99	12	72	4	96.1 (90.4–98.9)	85.7 (76.4–92.4)	6.7 (4.0–11.4)	0.05 (0.02–0.1)	89.2 (83.0–93.3)	94.7 (87.3–97.9)
>54	98	11	73	5	95.2 (89.0–98.4)	86.9 (77.8–93.3)	7.3 (4.2–12.6)	0.06 (0.02–0.1)	89.9 (83.7–93.9)	93.6 (86.1–97.2)
>56	98	10	74	5	95.2 (89.0–98.4)	88.1 (79.2–94.1)	7.9 (4.5–14.3)	0.06 (0.02–0.1)	90.7 (84.5–94.6)	93.7 (86.2–97.2)
>59	96	10	74	7	93.2 (86.5–97.2)	88.1 (79.2–94.1)	7.8 (4.4–14.0)	0.08 (0.04–0.2)	90.6 (84.3–94.5)	91.4 (83.7–95.6)
>61	96	8	76	7	93.2 (86.5–97.2)	90.5 (82.1–95.8)	9.8 (5.1–19.0)	0.08 (0.04–0.2)	92.3 (86.1–95.9)	91.6 (84.1–95.7)
>64	95	8	76	8	92.2 (85.3–96.6)	90.5 (82.1–95.8)	9.7 (5.0–18.8)	0.09 (0.04–0.2)	92.2 (86.0–95.8)	90.5 (83.0–94.9)
>68	93	8	76	10	90.3 (82.9–95.2)	90.5 (82.1–95.8)	9.5 (4.9–18.4)	0.11 (0.06–0.2)	92.1 (85.7–95.8)	88.4 (80.8–93.2)
>80	84	7	77	19	81.6 (72.7–88.5)	91.7 (83.6–96.6)	9.8 (4.8–20.0)	0.20 (0.1–0.3)	92.3 (85.4–96.1)	80.2 (72.9–85.9)
>90	76	6	78	27	73.8 (64.2–82.0)	92.9 (85.1–97.3)	10.3 (4.7–22.5)	0.28 (0.2–0.4)	92.7 (85.3–96.5)	74.3 (67.5–80.1)
>130	57	4	80	46	55.3 (45.2–65.1)	95.2 (88.3–98.7)	11.6 (4.4–30.7)	0.47 (0.4–0.6)	93.4 (84.4–97.4)	63.5 (58.3–68.4)
>171	41	2	82	62	39.8 (30.3–49.9)	97.6 (91.7–99.7)	16.7 (4.2–67.1)	0.62 (0.5–0.7)	95.3 (83.6–98.8)	56.9 (53.0–60.8)
>200	29	0	84	74	28.2 (19.7–37.9)	100.0 (95.7–100.0)	-	0.72 (0.6–0.8)	100.0	53.2 (50.1–56.2)

+LR, positive likelihood ratio; −LR, negative likelihood ratio; FN, false negatives; FP, false positives; +PV, positive predictive value; −PV, negative predictive value, SN, sensitivity; SP, specificity; TN, true negatives; TP, true positives.

**Table 5:** Sensitivity and specificity of different aldosterone cut-offs after saline infusion measured by LC-MS/MS.

ALD, ng/L	TP, *n*	FP,* n*	TN, *n*	FN, *n*	SN (95% CI)	SP (95% CI)	+LR (95% CI)	-LR (95% CI)	+PV (95% CI)	-PV (95% CI)
>47	93	22	62	10	90.3 (82.9–95.2)	73.8 (63.1–82.8)	3.5 (2.4–5.0)	0.13 (0.07–0.2)	80.9 (74.6–85.9)	86.1 (77.2–91.9)
>53	89	18	66	14	86.4 (78.2–92.4)	78.6 (68.3–86.8)	4.0 (2.7–6.1)	0.17 (0.1–0.3)	83.2 (76.5–88.2)	82.5 (74.1–88.6)
>56	86	18	66	17	83.5 (74.9–90.1)	79.8 (69.6–87.7)	4.1 (2.7–6.4)	0.21 (0.1–0.3)	83.5 (76.6–88.6)	79.8 (71.6–86.0)
>57	86	16	68	17	83.5 (74.9–90.1)	80.9 (70.9–88.7)	4.4 (2.8–6.9)	0.20 (0.1–0.3)	84.3 (77.4–89.4)	80.0 (71.9–86.2)
>59	85	15	69	18	82.5 (73.8–89.3)	84.5 (75.0–91.5)	5.3 (3.2–8.9)	0.21 (0.1–0.3)	86.7 (79.7–91.6)	79.8 (72.0–85.8)
>64	82	11	73	21	79.6 (70.5–86.9)	86.9 (77.8–93.3)	6.1 (3.5–10.6)	0.23 (0.2–0.3)	88.2 (81.0–92.9)	77.7 (70.2–83.7)
>68	82	9	75	21	79.6 (70.5–86.9)	89.3 (80.6–95.0)	7.4 (4.0–13.9)	0.23 (0.2–0.3)	90.1 (83.0–94.5)	78.1 (70.8–84.0)
>69	81	9	75	22	78.6 (69.5–86.1)	89.3 (80.6–95.0)	7.3 (3.9–13.7)	0.24 (0.2–0.3)	90.0 (82.8–94.4)	77.3 (70.0–83.3)
>70	80	8	76	23	77.7 (68.4–85.3)	90.5 (82.1–95.8)	8.2 (4.2–15.9)	0.25 (0.2–0.4)	90.9 (83.7–95.1)	76.8 (69.6–82.7)
>72	78	7	77	25	75.7 (66.3–83.6)	91.7 (83.6–96.6)	9.1 (4.4–18.6)	0.26 (0.2–0.4)	91.8 (84.5–95.8)	75.5 (68.5–81.3)
>77	73	6	78	30	70.9 (61.1–79.4)	92.9 (85.1–97.3)	9.9 (4.5–21.7)	0.31 (0.2–0.4)	92.4 (84.8–96.4)	72.2 (65.7–77.9)
>80	73	5	79	30	69.9 (60.1–78.5)	94.1 (86.7–98.0)	11.7 (5.0–27.7)	0.32 (0.2–0.4)	93.5 (85.9–97.1)	71.8 65.4–77.5)
>110	58	3	81	45	56.3 (46.2–66.1)	96.4 (89.9–99.3)	15.8 (5.1–48.5)	0.45 (0.4–0.6)	95.1 (86.3–98.3)	64.3 (59.0–69.2)
>140	44	2	82	59	42.7 (33.0–52.8)	97.6 (91.7–99.7)	17.9 (4.5–71.9)	0.59 (0.5–0.7)	95.7 (84.6–98.9)	58.2 (54.0–62.2)

ALD, aldosterone; FN, false negatives; FP, false positives; +LR, positive likelihood ratio; −LR, negative likelihood ratio, +PV, positive predictive value, −PV, negative predictive value, SN, sensitivity; SP, specificity; TN, true negatives; TP, true positives..

### Dynamics of steroid panel testing during SIT and its value for PA diagnosis

**Figure 5:**
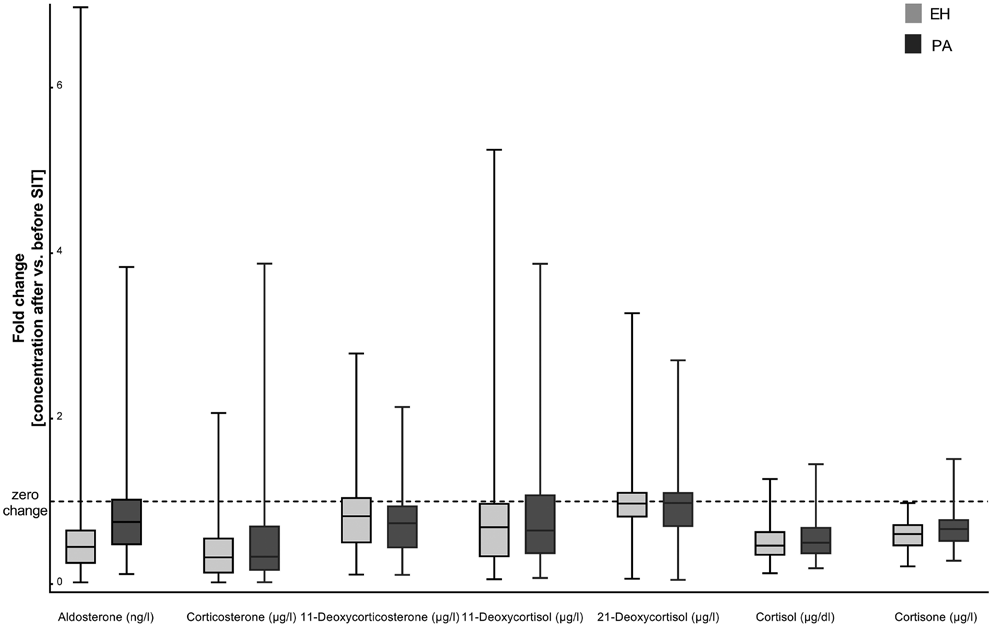
Fold changes (before vs after saline infusion) of steroids measured by LC-MS/MS during saline infusion testing in patients with essential hypertension (EH, *n* = 84) and primary aldosteronism (PA, *n* = 103). Dashed line: no change in steroid concentration before vs after saline infusion.

**Figure 6:**
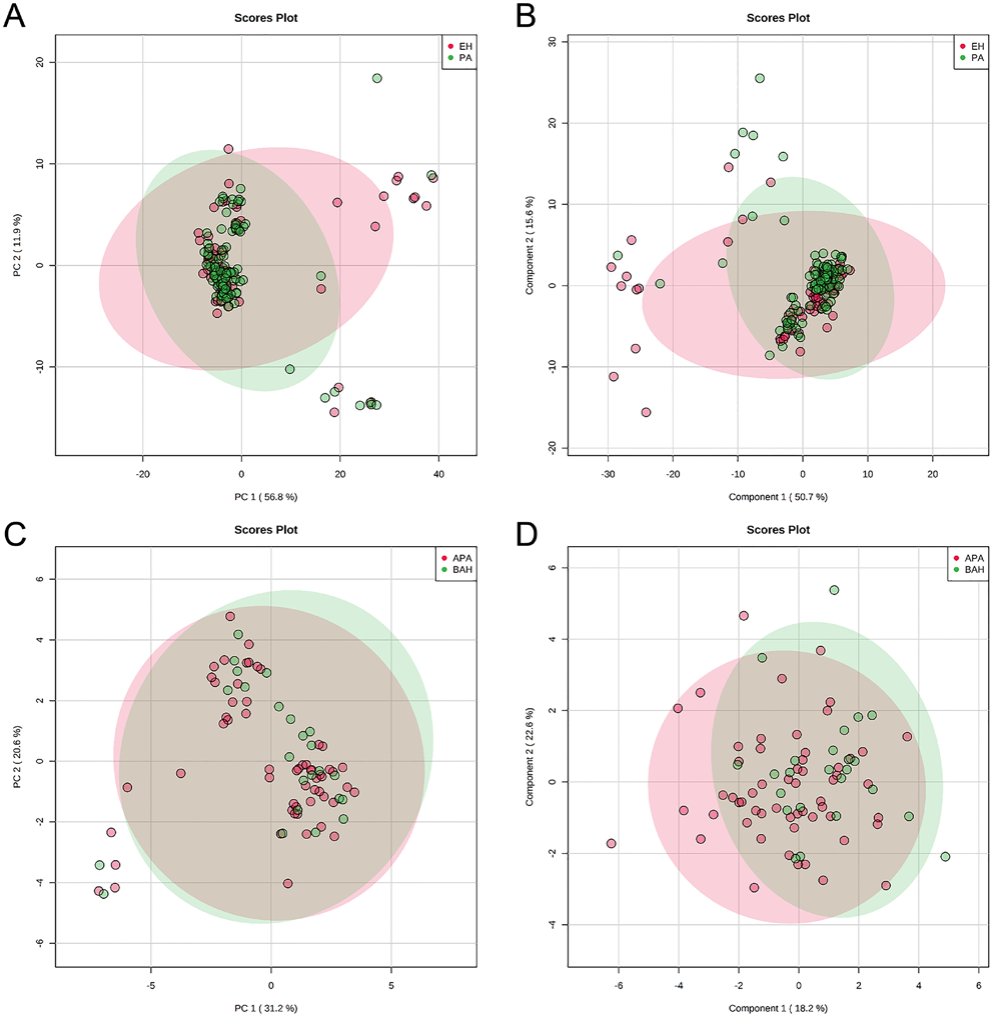
(A) Principal component analysis score plot for separation between primary aldosteronism (PA) and essential hypertension (EH) based on steroid profiles. (B) Partial least square – discriminant analysis score plot for separation between primary aldosteronism (PA) and essential hypertension (EH) based on steroid profiles. (C) Principal component analysis score plot for separation between unilateral (APA) and bilateral (BAH) primary aldosteronism based on steroid profiles. (D) Partial least square – discriminant analysis score plot for separation between unilateral (APA) and bilateral (BAH) primary aldosteronism based on steroid profiles.

To understand the dynamics of aldosterone precursors after saline infusion, we explored changes of 11-deoxycorticosterone, 21-deoxycortisol, 11-deoxycortisol, corticosterone, cortisol and cortisone before and after SIT (Fig. 5). We found a significant decrease of all analytes before vs after SIT (all *P* < 0.001) and statistically significant higher concentrations of 11-deoxycorticosterone in post-SIT samples from PA compared to EH patients (*P* < 0.001), whereas no differences in fold changes of all other steroids could be detected. Accordingly, none of these analytes was of similar diagnostic value as aldosterone alone. We furthermore performed principal compound analysis (PCA) and partial least square discriminant analysis (PLS-DA) for differentiation between PA and EH (Fig. 6A and B) as well as APA and BAH (Fig. 6C and D) based on steroid profiles after saline infusion and did not find improved discrimination by measurement of additional steroids.

## Discussion

The low positive predictive value of screening for PA using ARR leads to the necessity of confirmatory testing (1). Positive confirmatory test results pave the way for invasive workup and surgery which may cure patients with APA (31, 32). To date, no ‘gold standard’ for case confirmation of PA has been established due to inconclusive results of several studies regarding diagnostic accuracy of, for example, saline infusion, captopril challenge, or fludrocortisone suppression (9, 33, 34) and current guidelines do not express a specific recommendation for one particular test. By using oral sodium suppression and urinary aldosterone concentration as a measure of aldosterone exposure, it has been recently confirmed that there is a wide continuum of normal to relatively high and excessive aldosterone exposure in patients with hypertension (35). In our study we provide method-specific cut-offs that rely on the testing by IA and clinical judgment at the time of referral. Recumbent SIT is one of the most commonly used confirmatory tests in which serum aldosterone is measured after 4 h of saline infusion to detect aldosterone suppression or autonomous secretion (10) usually applying RIA or CLIA (14, 36). In the present work we evaluated highly sensitive measurement of aldosterone with different analytical techniques (i.e. LC-MS/MS, RIA and CLIA) and found – as already reported by others (17, 25, 27) – that aldosterone concentrations measured by LC-MS/MS were generally lower than those derived from IA. Of note, however, assay-specific cut-offs for LC-MS/MS and IA- based on clinical diagnosis of PA were similar.

Few studies have evaluated the utility of LC-MS/MS for diagnosis (27) and confirmatory testing (28) of PA. With a representative cohort of 187 cases (PA, *n* = 103; EH, *n* = 84), this is the first large series to study aldosterone during SIT by LC-MS/MS. Within a relatively short time interval, this analytical methodology has found its way into diagnostic routine especially for the quantification of androgens and precursors (37, 38, 39). Aldosterone is part of most established steroid panels and can be measured with sufficient sensitivity and precision to detect the low aldosterone concentrations present after saline infusion when state-of-the-art instrumentation and thorough validation is applied (10, 36, 40). IA-based analysis of aldosterone is afflicted with specific shortcomings such as varying antibody specificity and antibody cross-reactivity with structurally similar compounds which leads to overestimation of aldosterone (41). Previous studies reported significant variations between different IAs (12, 14, 16). In our study, IA measurement was on average 31 ng/L higher than LC-MS/MS quantification, and this is well in line with previous reports despite good correlation (28, 42, 43). Even by using highly sensitive and specific IAs like in this study, the lower limit of quantification is significantly higher in IA compared to LC-MS/MS. With the limits of quantification used here, aldosterone after SIT could be quantified in 372/374 samples with LC-MS/MS but only in 66/374 samples with IA. Optimal cut-off for LC-MS/MS of 69 ng/L reached a sensitivity of only 79% with a specificity of 89%. This is in contrast to a recently published paper by Fries *et al.*, in which the authors propose a lower LC-MS/MS cut-off for aldosterone after SIT compared to IA (44). To establish an LC-MS/MS specific cut-off for aldosterone during SIT the authors used two approaches sequentially: First, they developed a regression equation to determine LC-MS/MS cut-offs based on previously obtained IA results. Second, aldosterone was quantified by LC-MS/MS in samples from patients with PA and EH in their center and aldosterone suppression to <83 pmol/L – the LLOQ of that laboratory – was found in 92.5% of EH patients. The latter cut-off was then validated in samples from the German Conn Registry. This study as well as in the analysis by Guo *et al.* (28) used the Youden Index as the criterion for cut-off development which equally takes into account sensitivity and specificity. In the present study we chose to base our cut-off on the criterion of a positive predictive value of 90% rather than the Youden Index. Using a positive predictive value of 90%, the LC-MS/MS cut-off in the study by Fries *et al.* would have been higher by 37 pmol/L compared to the one proposed using the Youden Index. Finally, differences in the case definitions between the studies as well as the increased sensitivity of our method likely contribute to the differences between the published studies and ours.

Our analyses of the course of further steroid hormones during SIT do not add any diagnostic value in decision making, even though 11-deoxycorticosterone was significantly higher in PA after SIT than in EH. However, we did not measure 18-oxocortisol and 18-hydroxycortisol, both representing hybrid steroids that show promise regarding non-invasive subtype differentiation in PA (45, 46).

Despite the higher accuracy of LC-MS/MS measurement and associated lower LC-MS/MS test results, LC-MS/MS mis-classified nearly 17% of PA patients as unaffected by PA and falsely classified 5% of essential hypertensives as having PA. This is most likely explained by the fact that aldosterone values ≥50 ng/L in IA were used to trigger further evaluation for PA.

Two main features of PA need to be taken into account when considering confirmatory testing:

1. It is becoming increasingly clear that PA is part of a broad spectrum of disease ranging from physiologic, non-autonomous aldosterone secretion over the development of aldosterone producing cell clusters (47) to more severe forms of BAH and APAs. For the latter, disease-causing somatic mutations have been identified in the KCNJ5 (48), CACNA1D (49), ATP1A1 or ATP2B3 genes (50), which are clinically associated with a more severe phenotype and a higher prevalence of hypokalemia than in BAH (1). Specific subtypes of APA are associated with detectable concentrations of hybrid steroids 18-oxocortisol and 18-hydroxycortisol in peripheral blood and blood from AVS (46). They can be used diagnostically and are detectable at tissue level with mass spectrometry imaging (51) but most likely are synthesized at negligible quantities in non-APA PA and therefore are of limited value in cases of low or borderline aldosterone excess. The broad disease spectrum is well reflected by the curbed run of the LC-MS/MS ROC curve.
2. Current clinical decision making and consequent diagnosis of PA usually relies on one single measurement of aldosterone during SIT. Since clinical decision making in our series was based on clinical evaluation, imaging results and a single IA test result it is not surprising that some cases with aldosterone concentrations after SIT at or slightly above the cut-off of 50 ng/L underwent further workup and finally PA was diagnosed. It is conceivable that repeated testing might have excluded PA in these cases and conversely some apparent EH patients indeed in the course of disease may develop PA. Hence, the arbitrary cut-off of 50 ng/L confounds the seemingly more convincing results of IA.

The case definition is a limitation of our study. Indeed it would be desirable to clinically confirm diagnosis during follow-up and perform repeat testing in borderline cases. Therefore we cannot exclude potential bias due to exclusion of patients with potentially interfering medication, repeat testing or unclear diagnosis. However, in a clinical setting this is unrealistic. A further limitation to the broad applicability of LC-MS/MS as used in our study is the relatively sensitive equipment used for aldosterone quantification during SIT. This may be different at some institutions due to higher LLOQ associated with the use of different instrumentation and test setup. The most critical drawbacks of the present study are, however, (i) the determination of diagnosis of PA based on IA results and (ii) the absence of a separate validation cohort.

In conclusion, the ideal diagnostic criterion for PA remains to be established and LC-MS/MS alone might not be the solution. Our study provides the basis to use this emerging method in clinical routine. At our center, we will consider aldosterone by LC-MS/MS of 69 ng/L or higher to trigger further workup; in presence of lower results, we suggest to discuss the value of repeated testing with the patient to acknowledge the continuum of alterations in aldosterone secretion and the limited reliability of single hormone measurements. A prospective cohort with structured follow-up may help to decide which patients ultimately require complete PA workup and better define method-specific cut-offs for diagnosis.

## Supplementary Material

Supplementary Material 1

Supplementary Material 2

## Declaration of interest

Martin Fassnacht is a senior editor of the European Journal of Endocrinology. Martin Fassnacht was not involved in the peer review or editorial process for this paper on which he is listed as an author.

## Funding

This research did not receive any specific grant from any funding agency in the public, commercial or not-for-profit sector.

## Data availability

The datasets generated during and/or analyzed during the present study are not publicly available but are available from the corresponding author on reasonable request.

## Author contribution statement

Conception/planning of the work: Matthias Kroiss, Stefanie Hahner, Carmina Teresa Fuss, Max Kurlbaum, Martin Fassnacht; contribution of patient data and samples: Timo Deutschbein, Stefanie Hahner, Carmina Teresa Fuss, Max Kurlbaum, Martin Fassnacht; laboratory analyses: Max Kurlbaum, Matthias Kroiss, Sabine Kendl; data analysis: Katharina Brohm, Carmina Teresa Fuss, Max Kurlbaum, Anke Hannemann; nterpretation of results: Carmina Teresa Fuss, Matthias Kroiss, Martin Fassnacht, Timo Deutschbein, Max Kurlbaum, Stefanie Hahner, Anke Hannemann; writing of the first paper draft: Carmina Teresa Fuss, Matthias Kroiss. All authors contributed to the critical interpretation of the results, reviewed the manuscript for important intellectual content, approved the final version of the manuscript, and have agreed to be accountable for his/her role in this manuscript.
