# Supplementary material for: Confirmatory testing of primary aldosteronism with saline infusion test and LC-MS/MS
Source: Eur J Endocrinol. 2020 Oct 20;184(1):167–78. doi: 10.1530/EJE-20-0073 (PMC7709890; doi:10.1530/EJE-20-0073)
Supplement: Supplementary Material 1 [file supplementary_material_1.pdf]

Supplementary Material

Confirmatory Testing of Primary Aldosteronism with Saline Infusion Test and LC-MS/MS

Carmina Teresa Fuss[1], Katharina Brohm[1], Max Kurlbaum[1,2], Anke Hannemann[3], Sabine Kendl[1], Martin Fassnacht[1], Timo Deutschbein[1], Stefanie Hahner[1], Matthias Kroiss[1,2]

[1]Division of Endocrinology and Diabetes, Department of Internal Medicine I, University Hospital, University of Würzburg, Germany

[2]Central Laboratory, Core Unit Clinical Mass Spectrometry, University Hospital Würzburg , Germany

[3]Institute of Clinical Chemistry and Laboratory Medicine, University Medicine Greifswald, Germany

Short Title: LC-MS/MS-specific Cut-Off for Saline Infusion Test

Key Words: primary aldosteronism, mass spectrometry, salt loading test, Conn's syndrome, secondary arterial hypertension

Corresponding Author:

Matthias Kroiss, M.D., PhD

Department of Internal Medicine I

Endocrine and Diabetes Unit

University Hospital Würzburg

University of Würzburg

Oberdürrbacher Strasse 6

97080 Würzburg (Germany)

Phone: +49-(0)931-201-39740

Fax: +49-(0)931-201-6039740

E-Mail: Kroiss_M@ukw.de

Supplementary Figure 1

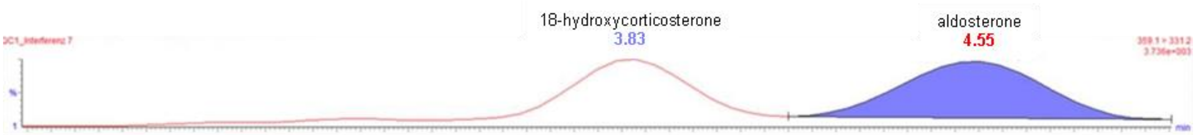

LC-MS/MS chromatogram demonstrating base-line separation between aldosterone and 18-hydroxycorticosterone. Retention time 18-hydroxycorticosterone = 3.83 min, retention time aldosterone = 4.55 min.

Supplementary Figure 2

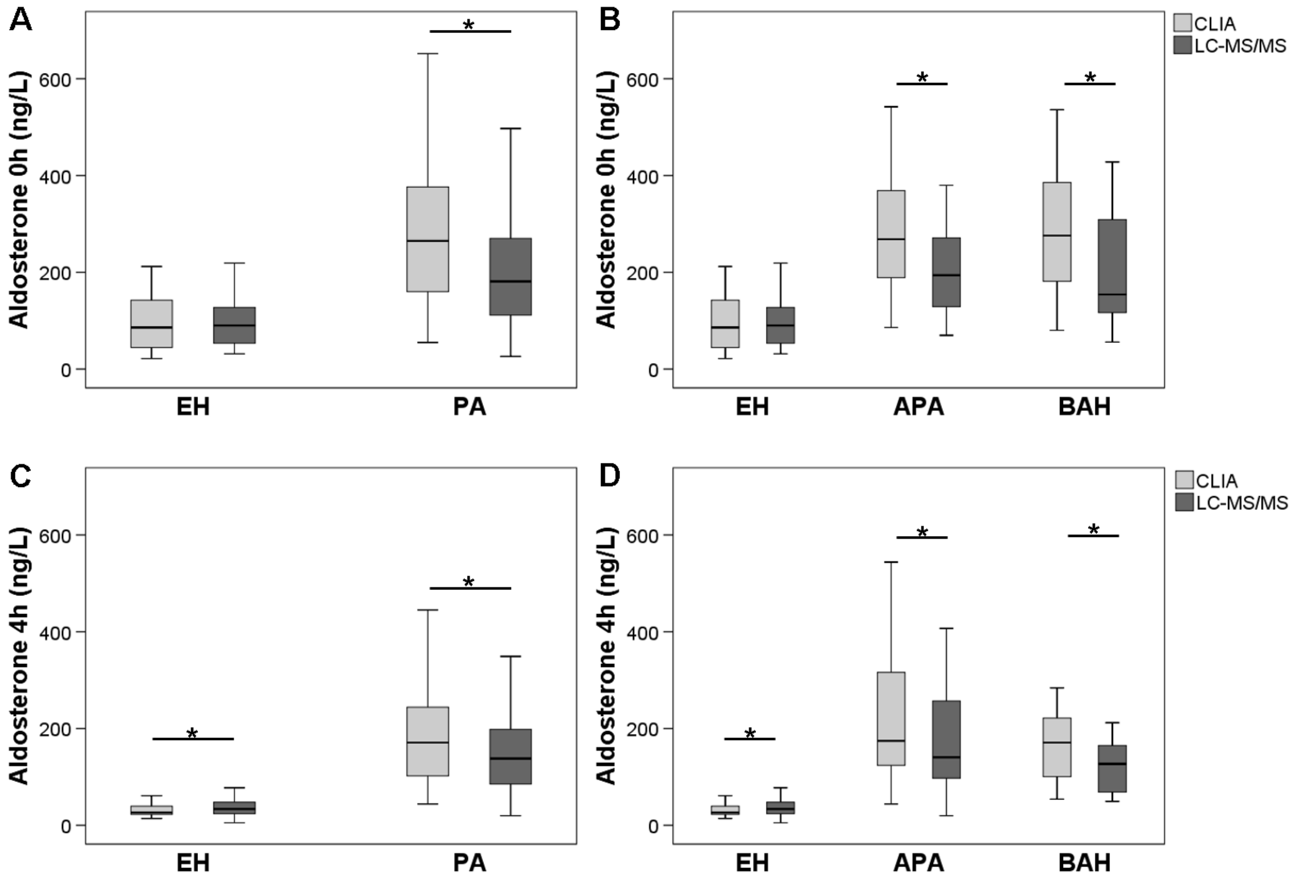

Aldosterone concentrations measured by **CLIA** (ISYS®) and LC-MS/MS during saline infusion testing in patients with essential hypertension (EH, **A-D**), primary aldosteronism (PA, **A+C**), aldosterone-producing adenoma (APA, **B+D**) and bilateral adrenal hyperplasia (BAH, **B+D**) before (0h, **A+B**) and after (4h, **C+D**) saline infusion. *p<0.01 (Wilcoxon test).

Supplementary Figure 3

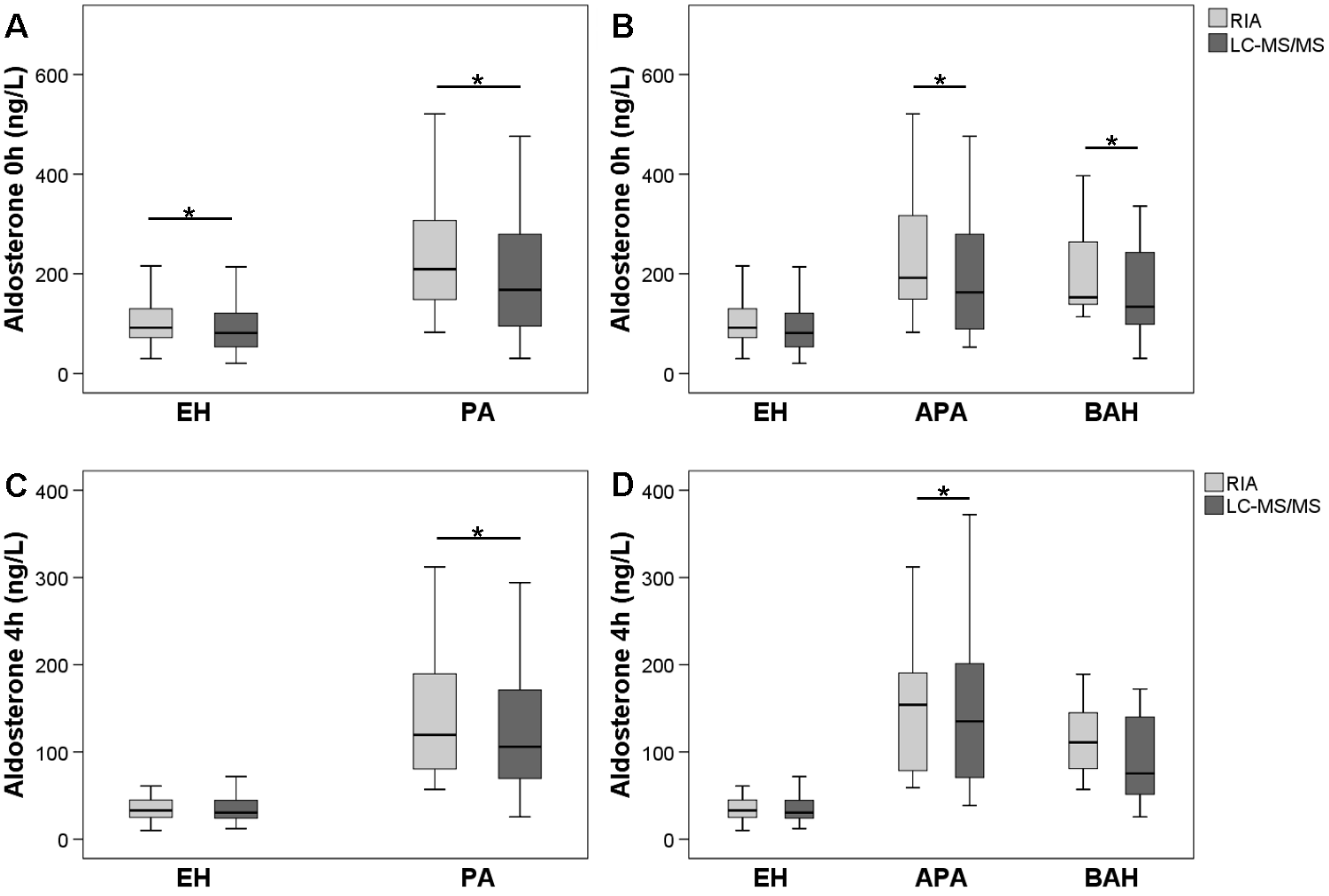

Aldosterone concentrations measured by **RIA** (Siemens®) and LC-MS/MS during saline infusion testing in patients with essential hypertension (EH, **A-D**), primary aldosteronism (PA, **A+C**), aldosterone-producing adenoma (APA, **B+D**) and bilateral adrenal hyperplasia (BAH, **B+D**) before (0h, **A+B**) and after (4h, **C+D**) saline infusion. *p<0.01 (Wilcoxon test).
